# Population-Based Multicentric Survey of Hepatitis B Infection and Risk Factors in the North, South, and Southeast Regions of Brazil, 10–20 Years after the Beginning of Vaccination

**DOI:** 10.4269/ajtmh.15-0216

**Published:** 2015-12-09

**Authors:** Ricardo A. A. Ximenes, Gerusa M. Figueiredo, Maria Regina A. Cardoso, Airton T. Stein, Regina C. Moreira, Gabriela Coral, Deborah Crespo, Alex A. dos Santos, Ulisses R. Montarroyos, Maria Cynthia Braga, Leila M. M. B. Pereira, for the Hepatitis Study Group

**Affiliations:** Hospital Universitário Oswaldo Cruz, Faculdade de Ciências Médicas de Pernambuco, Universidade de Pernambuco, Pernambuco, Brazil; Departamento de Medicina Tropical, Universidade Federal de Pernambuco, Cidade Universitária, Pernambuco, Brazil; Instituto de Medicina Tropical da Universidade de São Paulo, São Paulo, Brazil; Departamento de Epidemiologia, Faculdade de Saúde Pública, Universidade de São Paulo, São Paulo, Brazil; Fundação Universidade Federal de Ciências da Saúde de Porto Alegre, Rio Grande do Sul, Brazil; Instituto Adolfo Lutz, São Paulo, Brazil; Secretaria de Saúde Pública do Estado do Pará, Pará, Brazil; Instituto Bioestatístico, Pará, Brazil; Instituto de Ciências Biológicas, Universidade de Pernambuco, Pernambuco, Brazil; Fundação Oswaldo Cruz, Centro de Pesquisas Aggeu Magalhães, Cidade Universitária, Pernambuco, Brazil; Instituto do Fígado de Pernambuco, Pernambuco, Brazil

## Abstract

A population-based hepatitis survey was carried out to estimate the prevalence of hepatitis B virus (HBV) infection and its predictive factors for the state capitals from the north, south, and southeast regions of Brazil. A multistage cluster sampling was used to select, successively, census tracts, blocks, households, and residents in the age group 10–69 years in each state capital. The prevalence of hepatitis B surface antigen (HBsAg) was lower than 1% in the north, southeast, and south regions. Socioeconomic condition was associated with HBV infection in north and south regions. Variables related to the blood route transmission were associated with HBV infection only in the south whereas those related to sexual behavior were associated with HBV infection in the north and south regions. Drug use was associated in all regions, but the type of drug differed. The findings presented herein highlight the diversity of the potential transmission routes for hepatitis B transmission in Brazil. In one hand, it reinforces the importance of national control strategies of large impact already in course (immunization of infants, adolescents, and adults up to 49 years of age and blood supply screening). On the other hand, it shows that there is still room for further control measures targeted to different groups within each region.

## Introduction

Hepatitis B virus (HBV) infection is still a major public health problem worldwide.^1^ Around 90% of infants infected perinatally become chronic carriers, unless vaccinated at birth. Approximately 25% of persons who became chronically infected during childhood and 15% of persons chronically infected after childhood die of cirrhosis or liver cancer.^2^

In 1989, the Brazilian government implemented HBV immunization for infants in the western area of the Amazonas State because of the high prevalence, and gradually expanded the program to the other northern states. In 1993, states in south and southeast regions extended immunization up to the age of 4 years. In 1998, the HBV vaccine was incorporated into the immunization schedule for infants as a national policy.^3^ Subsequently, children and adolescents (2001), young adults up to 29 years (2011) of age, and adults up to 49 years (2013) of age were included. Vaccination of risk groups started in 1992, and new groups were successively added to the original list.

A population-based survey conducted in 27 Brazilian cities among 17,749 children aged 18–30 months in 2007 estimated that 86.7% completed the three-dose series by 12 months of age.^4^

Most of the Brazilian territory has been classified as having intermediate and low endemicity for HBV infection, the exception being the high-HBV endemicity area located in the western Amazon region and restricted areas of some states in the south and southeast regions.^5^

A national population-based hepatitis survey was thus conducted aiming at estimating the prevalence of HBV infection and its predictive factors after about 10–20 years of the beginning of vaccination. Here we report the results for all the state capitals from the north, south, and southeast regions. Results showing low prevalence in the northeast and central-west regions and in the Federal District of Brasília have already been published.^6^

## Materials and Methods

### Study populations and study samples.

The Brazilian National Hepatitis A, B, and C Survey is a cross-sectional household study in a representative sample of the population resident in all the state capitals and in the federal district.

**Figure 1.**
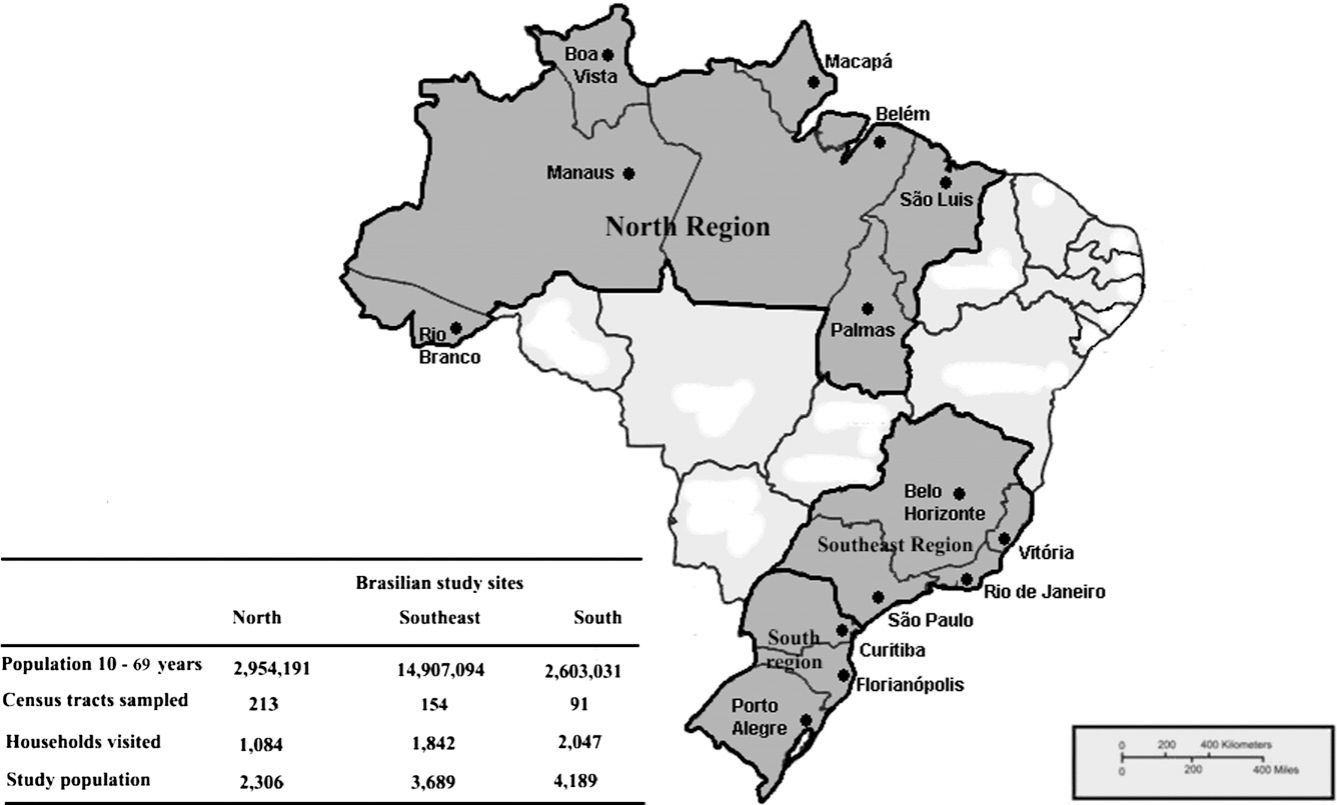
Distribution of population aged 13–69 years in the state capitals of the North, South and Southeast Regions of Brazil and of the census tracts, households and individuals sampled.

We report on the hepatitis B survey conducted in the southeast (four capital cities), south (three capital cities), and north (seven capital cities) regions, during March 2007–January 2008 (Figure 1

). The target population (10–69 years of age) comprised 14.9 million inhabitants in the southeast region, 2.6 million in the south, and 2.9 million in the north region.

More detailed information on sampling and data collection has already been reported.^7^ In brief, the study population was stratified into two age strata (10–19 and 20–69 years) and by state capital in each region; the samples for each age group were allocated among the state capitals using a constant sampling fraction. A random sample was obtained using a multistage cluster sampling strategy based on census tract, which is the smallest geographic unit with sociodemographic data available (approximately 300 households corresponding to 1,000 inhabitants). To improve sampling efficiency, the census tracts were, when necessary, either broken up or grouped together according to preestablished criteria to make the primary sampling unit (PSU). The PSUs were then ordered according to the number of years of schooling of the head of household, and a systematic sample was taken from this ordered list of PSUs, with probability proportional to size, so that all socioeconomic groups would be represented. In the second stage, blocks were systematically selected with probability proportional to size within each PSU and, finally, a systematic sampling of households was drawn in each block. All residents in the age group considered were recruited in the selected households. Number of years of schooling of the head of household was selected to order the census tracts because it is strongly correlated with income.

A total of 10,184 individuals were enrolled in the study: 3,689 persons in 1,842 households living in 154 census tract in the southeast region; 4,189 persons in 2,047 households located in 91 census tracts in the south region; and 2,306 persons in 1,084 households living in 213 census tracts in the north region.

#### Data collection.

Data on socioeconomic status and potential risk factors were collected by trained interviewers using a structured questionnaire during household visits. Self-reported HBV vaccine status was assessed and confirmed by the vaccination card, when available.

#### Laboratory tests and definitions.

Blood samples were collected after the interview and tested for antibodies against hepatitis B core antigen (HBc) using an enzyme-linked immunosorbent assay (AxsymTM; Abbott Laboratories, Abbott Park, IL) in central public health laboratories. Positive samples were tested for antibodies against hepatitis B surface antigen (HBs); samples negative for antibodies against hepatitis B surface antigen (anti-HBs) were tested for hepatitis B surface antigen (HBsAg), using the same technique (AxsymTM). Borderline results were retested and indeterminate results were excluded from the analysis.

Chronic HBV infection was determined as the presence of HBsAg and anti-HBc. The presence of HBV infection, past or present, was determined by detection of anti-HBc, in accordance with the National Health and Nutritional Examination Survey.^8^

#### Statistical analysis.

Prevalence and 95% confidence intervals (95% CI) were corrected by the design effect. Risk factors comprised questions on drug use and sexual practice, and therefore the analysis included only persons aged 13–69 years. Association between HBV infection and individual variables, adjusted by age, was assessed by odds ratio (OR) with a 95% CI. Variables associated in bivariate analyses with *P* < 0.20 were successively included in a multivariate logistic regression model. ORs were corrected using the random design effect caused by the study design and weighting caused by the difference in sampling fractions between age groups.

For participants who presented a vaccination card, the predictive positive and negative values of the self-reported HBV vaccination status were estimated.

Stata, version 9.2 (College Station, TX), was used for statistical analysis.

#### Sample size.

Details on sample size and sampling procedures have already been published.^7^

#### Ethical issues.

Interviews and blood sample collection were performed after a written informed consent was signed. For minors, the legal guardian consent was obtained. The project was approved by the National Research Ethics Committee of the Brazilian National Health Council and by the local research ethics committees.

Each positive result was delivered by the medical coordinator, and all negative results were sent by mail to participants. Individuals who were positive for HBsAg were referred to hepatology or infectology outpatient clinics in public referral hospitals for clinical evaluation. Persons negative for HBV were referred to public health services for vaccination.

## Results

### Prevalence.

The prevalence of anti-HBc stratified according to region and age group is shown in Table 1. The prevalence in the age group 10–19 years was higher in the south whereas that in the age group 20–69 years was higher in the north.

A total of 15, 14, and 21 carriers were identified in the north, southeast and south, respectively.

Vaccination status was reported by approximately 78% of the population in all regions. Of the 3,338 participants who presented a vaccination card, the predictive positive and negative values of the self-reported HBV vaccination status were 90.38% (95% CI: 87.03–93.10%) and 75.47% (95% CI: 61.72–86.24%) for the north, 95.97% (95% CI: 94.50–97.13%) and 96.21% (95% CI: 91.38–98.76%) for the south, and 95.67% (95% CI: 94.11–96.92%) and 89.45% (95% CI: 84.59–93.19%) for the southeast region. Approximately 45.6% reported HBV vaccination. Residents of the north region reported higher frequencies of HBV vaccination (56%) compared with those in the south (41%) and southeast (40%) regions. Vaccination status for persons aged ≥ 20 years was significantly higher in the north compared with the south and southeast regions (Table 1).

#### Bivariate analysis.

Tables 2–5 show the association of individual risk factors with HBV infection, adjusted by age. For sexual behavioral factors, in the bivariate analysis, the reference category was that with lower potential of transmission (among those who had initiated sexual life), whereas in the multivariate, the reference was those who had not started sexual life. In the north region, having a bisexual partner, another sexual partner besides current partner, and use of smoked drugs (marijuana, crack cocaine) were statistically associated with HBV infection. In the southeast, male gender, presence of tattoo, use of smoked or inhaled drugs (shoe glue, toluene), and heavy alcohol consumption were all associated with HBV infection. In the south, the characteristics associated with HBV infection were blood transfusion in the past 12 months, sharing toothbrush, initiated sexual life, no use of condom, previous sexually transmitted disease (STD), another sexual partner besides current partner, and use of inhaled, sniffed (cocaine), or injected drugs.

#### Multivariable analysis.

The multivariate model showed that age was an independent risk factor in all regions (Table 6). Socioeconomic condition was associated with HBV infection in north and south regions. Variables related to the blood route transmission were associated with HBV infection only in the south whereas those related to sexual behavior were associated in the north and south regions. Drug use was associated in all regions, but the type of drug differed. The south was the region with a wider spectrum of risk factors.

## Discussion

Knowledge of region- and age-specific prevalence of hepatitis B infection is important for evaluating vaccination programs and national disease prevention and control efforts. Our findings classify these regions as having low HBV endemicity, rather than the intermediate and high endemicity defined by previous studies in the last century and past decade. Nevertheless, the overall estimation of individuals chronically infected with HBV was 18,612, 43,367, and 12,624 in the north, southeast, and south, respectively.

The introduction of vaccination in these regions happened in different years and in different age groups. In the data collection period, vaccinated cohorts might have reached the age of 20 years. Note that in the north, area that was considered as having high prevalence, the vaccination status for participants over 20 years of age was significantly higher compared with the south and southeast regions.

Countries with high prevalence of HVB infection that began universal immunization have shown decreased prevalence, as occurred in Taiwan,^9^,^10^ China,^11^,^12^ and Saudi Arabia.^13^ A reduction was also observed in low-endemicity countries, such as the United States^14^ and Canada.^15^

In Brazil, a population-based study in the western Amazon State, in a region called Labrea (where the association of hepatitis B and D virus infection was first identified), revealed a significant reduction in the HBsAg rate in the community, 11 years after the introduction of the HBV vaccination program, categorizing the area as intermediate endemicity (formerly classified as high).^16^

Our data revealed a prevalence of HBsAg similar to the data for all capitals of the northeast, central-west regions, and in the federal capital.^6^

Although population-based data for HBV infection before and after vaccination are not available for large areas, the low-endemicity level found strongly suggest an impact of the vaccination policy. The Brazilian National Immunization Program attained high vaccination coverage and sustainability as vaccines were freely supplied by the National Self-Sufficiency Program in Immunobiologicals.^17^

Recent publication with the objective of estimating age-, sex-, and region-specific prevalence of chronic HBV infection in the world and calculate the absolute number of persons chronically infected, using an empirical Baysian hierarchical model, showed that from 1990 to 2005 the prevalence of chronic HBV infection decreased in most regions. Brazil was categorized as a region of low endemicity.^18^

The results of this survey indicate different patterns of HBV transmission in the areas studied. The factors independently associated with HBV infection in the north region were mainly related to the sexual route and to the use of drugs; in the southeast to the use of drugs; while in the south to the sexual route, use of drugs and also to the blood route. An inverse relationship with socioeconomic conditions (schooling) was found in the north and south regions. Use of drugs that were not related to the mechanism of transmission of HBV was also associated with seropositivity.

Age was an independent predictor for HBV infection, as reported in the literature,^19^ indicating length of time for viral exposures and initiation of sexual activity. The increasing vaccine coverage of younger age groups may also have contributed to the difference in prevalence between the two age groups considered (10–19 and 20–69 years). Our study found higher risk of HBV infection in men only in the southeast region, like in the United States^20^ and in Brazilian studies with individuals recruited from restricted areas, volunteers, or outpatient clinics.^21^–^23^

The sexual behavior associated with exposure to HBV was not the same in the different regions. In the north, there was a greater chance of HBV infection for those whose partner had sex with individuals of the same sex, similar to what was found in the northeast.^6^ Male–male sexual activity^24^,^25^ and having a homosexual partner^26^ have been reported as risk factors for HBV infection in other studies. Individuals whose partners have sex with individuals of the same sex share, to some extent, the same risk factors of their partners. The association between the number of sexual partners and the positivity to serologic markers of hepatitis B (and other STDs) is often referred in Brazilian studies carried out either in blood centers^27^,^28^ or in general population^29^ or, still, in special groups.^30^,^31^

These sexual behaviors, found in the north and south regions, were probably related to unsafe sexual practices. The history of STD also expresses these practices. The Brazilian Ministry of Health has conducted several campaigns to promote responsible sexual behavior and has made condoms largely available.^32^ Further efforts could be done to deliver interventions and to conduct in-depth studies to target groups until the age group with high anti-HBV vaccination coverage gets older.

In the southeast, HBV infection was associated with the use of injected drugs; this association is well documented in the literature and in Brazilian studies.^25^,^33^ In the three regions, the use of non-injecting drugs was also associated with the presence of HBV infection. It is likely that in these settings non-injecting drug users are more prone to other risk behavior, which should be investigated to allow the choice of targeted control measures. The same rationale could be used to explain the association with heavy alcohol consumption in the southeast.

Sharing toothbrush was associated with HBV infection, inexplicably, in the region with higher Human Development Index. The association with history of blood transfusion and gastrointestinal endoscopy in the south should be interpreted with caution. As in Brazil the blood supply is adequately screened for hepatitis B since 1975, it is possible that either the transfusion occurred before this year or that the exposure to HBV occurred before the transfusion. Endoscopy-related transmission of HBV is rare.^34^ Quasi-experimental designs (before and after studies) could be performed to elucidate if blood transfusion and endoscopy still play any role in HBV transmission in southern Brazil.

Schooling was associated with exposure to HBV in the south region. Risk behaviors related to the socioeconomic context could be identified to guide specific interventions. Sewage disposal was associated with HBV infection in the north and south regions, but the association was in opposite directions suggesting that the same situation (household with septic tank) may express different socioeconomic conditions in these two regions. The results for hepatitis C for the whole country showed that no sewage disposal, a marker of extreme poverty, was associated with anti-hepatitis C virus prevalence.^35^

The study has some limitations and strengths. The main strength is that it is a representative sample of the population aged 10–69 years of the ensemble of state capitals in the north, south, and southeast regions. However, the results may not be directly extrapolated to the residents of smaller cities or rural areas. Another limitation is temporal ambiguity, which is inherent to cross-sectional studies. Information bias, especially in relation to questions related to the use of drugs and sexual behavior, may have occurred but, as individuals did not know the results of their blood testing when interviewed, it was nonrandom, which may have decreased the magnitude of some of the associations found.

The findings presented herein highlight the diversity of the potential transmission routes for hepatitis B transmission in Brazil. In one hand, it reinforces the importance of national control strategies of large impact already in course such as the immunization of infants, adolescents, and adults up to 49 years of age and blood supply screening. On the other hand, it shows that there is still room for further control measures targeted to different groups within each region.

## Figures and Tables

**Table 1:** Prevalence of hepatitis B antibodies, percentage of individuals vaccinated for hepatitis B, and estimated population ever infected in a representative sample of individuals living in the state capitals of Brazil, 2005–2009

Setting age (years) group	Participants	Prevalence* % (95% CI)	Vaccination status (%)	State capitals population	Estimated persons ever infected *n* (95% CI)
North					
10–19	1,157	1.1 (0.9–1.4)	68.9	860,922	9,471 (7,749–12,053)
20–69	1,149	11.6 (10.7–12.4)	49.8	2,093,269	242,820 (223,980–259,356)
Southeast					
10–19	1,794	0.61 (0.27–0.95)	67.4	3,295,110	20,100 (8,897–31,304)
20–69	1,867	7.90 (6.60–9.19)	23.2	11,661,984	921,297 (769,691–1,071,737)
South					
10–19	1,801	1.58 (0.83–2.32)	76.2	586,270	9,264 (4,867–13,602)
20–69	2,373	11.3 (9.94–12.7)	24.0	2,043,761	230,944 (203,150–259,558)

CI = confidence interval.

* Prevalence adjusted for random effect.

**Table 2:** Relative odds of hepatitis B infection for individual factors among persons of 13–69 years of age in three regions in Brazil, 2005–2009

Individual factors	North			Southeast			South		
	No.	OR (95% CI)*	*P* value	No.	OR (95% CI)*	*P* value	No.	OR (95% CI)*	*P* value
Sex									
Female	1,094	1.0	–	1,739	1.0	–	1,977	1.0	–
Male	875	1.39 (1.01–1.91)	0.041	1,368	1.60 (1.13–2.26)	0.007	1.650	1.19 (0.94–1.50)	0.141
Literacy									
Yes	1,909	1.0	–	3,009	1.0	–	3,536	1.0	–
No	56	2.93 (1.47–5.84)	0.002	98	0.67 (0.31–1.42)	0.303	91	0.95 (0.53–1.69)	0.870
Schooling									
Illiterate	112	1.0		190	1.0		180	1.0	
Basic level	797	0.61 (0.37–0.99)	0.047	1,389	1.06 (0.62–1.79)	0.823	1,404	0.81 (0.55–1.19)	0.293
Secondary level	814	0.59 (0.32–1.05)	0.075	1,172	0.84 (0.45–1.54)	0.578	1,428	0.58 (0.37–0.90)	0.016
University	217	0.34 (0.16–0.71)	0.004	334	0.47 (0.21–1.07)	0.074	580	0.41 (0.24–0.70)	0.001
Paid work past week									
Yes	813	1.0	–	1,420	1.0	–	1,839	1.0	–
No	1,156	0.54 (0.40–0.73)	0.000	1,687	0.86 (0.60–1.23)	0.420	1,787	1.00 (0.77–1.31)	0.966
Hospitalization									
Never	1,203	1.0	–	2,216	1.0	–	2,458	1.0	–
Past 12 months	182	0.77 (0,42–1,40)	0.388	168	1.02 (0.47–2.20)	0.948	189	1.27 (0.80–2.02)	0.307
Ever	559	1.04 (0.69–1.56)	0.847	718	1.07 (0.70–1.64)	0.726	979	1.26 (0.98–1.62)	0.071
Blood transfusion									
Never	1,815	1	–	2,894	1.0	–	3,289	1.0	–
Past 12 months	44	1.45 (0.50–4.20)	0.494	40	1.41 (0.51–3.92)	0.505	45	2.57 (1.36–4.86)	0.004
Ever	62	1.65 (0.82–3.32)	0.157	136	1.45 (0.72–2.94)	0.294	282	0.78 (0.49–1.23)	0.296
Surgery									
Never	1,177	1	–	1,725	1.0	–	1,754	1.0	–
Past 12 months	180	0.98 (0.58–1.65)	0.946	270	1.39 (0.82–2.34)	0.211	358	1.23 (0.79–1.90)	0.347
Ever	591	0.71 (0.49–1.02)	0.066	1,109	1.24 (0.80–1.92)	0.322	1,504	0.97 (0.72–1.30)	0.852

CI = confidence interval; OR = odds ratio.

* Weighted ORs adjusted for random effect and age.

**Table 5:** Relative odds of hepatitis B infection for drug use–related factors among persons of 13–69 years of age in three regions in Brazil, 2005–2009

Drug use-related factors	North			Southeast			South		
	No.	OR (95% CI)*	*P* value	No.	OR (95% CI)*	*P* value	No.	OR (95% CI)*	*P* value
Ever used smoked drugs									
No	1,651	1.0	–	2,814	1.0	–	3,038	1.0	–
Yes	191	1.73 (1.19–2.55)	0.005	290	1.72 (1.08–2.73)	0.022	570	1.31 (0.87–1.97)	0.187
Ever used inhaled drugs									
No	1,902	1.0	–	3,068	1.0	–	3,512	1.0	–
Yes	35	1.67 (0.75–3.67)	0.204	31	4.98 (1.60–15.5)	0.006	90	2.39 (1.13–5.05)	0.022
Ever used sniffed drugs									
No	1,877	1.0	–	2,969	1.0	–	3,392	1.0	–
Yes	71	2.75 (1.28–5.87)	0.009	134	1.53 (0.74–3.16)	0.241	216	1.95 (1.14–3.31)	0.014
Ever used injected drugs									
No	1,922	1.0	–	3,084	1.0	–	3,577	1.0	–
Yes	9	–	–	13	1.41 (0.24–8.09)	0.695	21	3.55 (1.31–9.61)	0.013
Previous use of glass syringe									
No	1,604	1.0	–	2,627	1.0	–	2,753	1.0	–
Yes	316	0.73 (0.47–1.15)	0.178	444	0.78 (0.54–1.12)	0.186	824	1.19 (0.89–1.58)	0.223
Alcohol consumption									
None	1,368	1.0	–	1,968	1.0	–	2,077	1.0	–
Light	472	0.87 (0.61–1.25)	0.455	978	1.10 (0.74–1.63)	0.627	1,364	1.02 (0.77–1.34)	0.878
Heavy	141	1.66 (0.92–2.98)	0.093	159	2.32 (1.31–4.09)	0.004	176	1.37 (0.75–2.51)	0.296

CI = confidence interval; OR = odds ratio.

* Weighted ORs adjusted for random effect and age.

**Table 6:** Factors associated with hepatitis B infection in three regions of Brazil, 2005

Characteristics	North		Southeast		South	
	OR (95% CI)*	*P* value	OR (95% CI)	*P* value	OR (95% CI)*	*P* value
Sex						
Female	–	–	1.0	–	–	–
Male	–	–	1.49 (1.02–2.19)	0.041	–	–
Age (in years)	1.05 (1.03–1.06)	0.000	1.06 (1.05–1.08)	0.000	1.04 (1.03–1.05)	0.000
Read and write						
Yes	1.0	–	–	–	–	–
No	3.14 (1.53–6.46)	0.002	–	–	–	–
Schooling						
Illiterate	–	–	–	–	1.0	–
Basic level	–	–	–	–	0.78 (0.53–1.15)	0.214
Secondary level and university	–	–	–	–	0.51 (0.32–0.81)	0.004
Paid work past week						
Yes	1.0	–	–	–	–	–
No	0.56 (0.40–0.76)	0.000	–	–	–	–
Sewage disposal						
Pubic system	1.0	–	–	–	1.0	–
Septic tanks	0.61 (0.40–0.93)	0.021	–	–	1.71 (1.28–2.29)	0.000
Other destination	0.83 (0.40–1.72)	0.617	–	–	–	–
Blood transfusion						
Never	–	–	–	–	1.0	–
Past 12 months	–	–	–	–	2.28 (1.11–4.67)	0.024
Ever	–	–	–	–	0.68 (0.41–1.15)	0.152
Endoscopy						
Never	–	–	–	–	1.0	–
Past 12 months	–	–	–	–	1.27 (0.80–2.05)	0.311
Ever	–	–	–	–	1.33 (0.99–1.80)	0.059
Share toothbrush						
No	–	–	–	–	1.0	–
Yes	–	–	–	–	1.81 (1.05–3.12)	0.032
Condom use						
Not started sexual life	–	–	–	–	1.0	–
Yes, regularly	–	–	–	–	2.41 (0.96–6.06)	0.060
Yes, sometimes	–	–	–	–	3.68 (1.65–8.22)	0.001
Never	–	–	–	–	3.90 (1.54–9.88)	0.004
Bisexual partner						
Not started sexual life	1.0		–	–	–	–
No	2.37 (0.87–6.42)	0.089	–	–	–	–
Yes	1.68 (1.17–2.41)	0.005	–	–	–	–
Ever used smoked drugs						
No	1.0	–	–	–	–	–
Yes	1.58 (1.05–2.36)	0.026	–	–	–	–
Ever used inhaled drugs						
No	–	–	1.0	–	–	–
Yes	–	–	3.85 (1.19–12.4)	0.024	–	–
Ever sniffed drugs						
No	–	–	–	–	1.0	–
Yes	–	–	–	–	1.73 (1.01–2.97)	0.048
Ever injecting drugs						
No	–	–	–	–	1.0	–
Yes	–	–	–	–	2.49 (0.82–7.54)	0.106
Classification elitism						
Abstemious	–	–	–	–	–	–
Light weight drinker	–	–	0.95 (0.63–1.44)	0.815	–	–
Heavy drinker	–	–	1.96 (1.06–3.59)	0.030	–	–

CI = confidence interval; OR = odds ratio.

* Adjusted ORs corrected for random effect and weighted for age group.
